# An Intense Out-of-Season Rebound of Influenza Activity After the Relaxation of Coronavirus Disease 2019 Restrictions in Beijing, China

**DOI:** 10.1093/ofid/ofae163

**Published:** 2024-03-20

**Authors:** Li Zhang, Wei Duan, Chunna Ma, Jiaojiao Zhang, Ying Sun, Jiaxin Ma, Yingying Wang, Daitao Zhang, Quanyi Wang, Jue Liu, Min Liu

**Affiliations:** Department of Epidemiology and Biostatistics, School of Public Health, Peking University, Beijing, China; Institute for Infectious Disease and Endemic Disease Control, Beijing Center for Disease Prevention and Control, Beijing, China; Institute for Infectious Disease and Endemic Disease Control, Beijing Center for Disease Prevention and Control, Beijing, China; Institute for Infectious Disease and Endemic Disease Control, Beijing Center for Disease Prevention and Control, Beijing, China; Institute for Infectious Disease and Endemic Disease Control, Beijing Center for Disease Prevention and Control, Beijing, China; Institute for Infectious Disease and Endemic Disease Control, Beijing Center for Disease Prevention and Control, Beijing, China; Institute for Infectious Disease and Endemic Disease Control, Beijing Center for Disease Prevention and Control, Beijing, China; Institute for Infectious Disease and Endemic Disease Control, Beijing Center for Disease Prevention and Control, Beijing, China; Institute for Infectious Disease and Endemic Disease Control, Beijing Center for Disease Prevention and Control, Beijing, China; Center Office, Beijing Center for Disease Prevention and Control, Beijing, China; Beijing Research Center for Respiratory Infectious Diseases, Beijing, China; Department of Epidemiology and Biostatistics, School of Public Health, Peking University, Beijing, China; Department of Epidemiology and Biostatistics, School of Public Health, Peking University, Beijing, China

**Keywords:** COVID-19, incidence, influenza, instantaneous reproduction number, surveillance

## Abstract

**Background:**

The aim of this study was to investigate the changes of epidemic characteristics of influenza activity pre– and post–coronavirus disease 2019 (COVID-19) in Beijing, China.

**Methods:**

Epidemiologic data were collected from the influenza surveillance system in Beijing. We compared epidemic intensity, epidemic onset and duration, and influenza transmissibility during the 2022–2023 season with pre-COVID-19 seasons from 2014 to 2020.

**Results:**

The overall incidence rate of influenza in the 2022–2023 season was significantly higher than that of the pre-COVID-19 period, with the record-high level of epidemic intensity in Beijing. The onset and duration of the influenza epidemic period in 2022–2023 season was notably later and shorter than that of the 2014–2020 seasons. Maximum daily instantaneous reproduction number (R_t_) of the 2022–2023 season (R_t_ = 2.31) was much higher than that of the pre-COVID-19 period (R_t_ = 1.49). The incidence of influenza A(H1N1) and A(H3N2) were the highest among children aged 0–4 years and 5–14 years, respectively, in the 2022–2023 season.

**Conclusions:**

A late, intense, and short-term peak influenza activity was observed in the 2022–2023 season in Beijing. Children <15 years old were impacted the most by the interruption of influenza circulation during the COVID-19 pandemic. Maintaining continuous surveillance and developing targeted public health strategies of influenza is necessary.

The emergence of coronavirus disease 2019 (COVID-19) in early 2020 has resulted in stringent public health measures worldwide, such as mask-wearing, hand-washing, social distancing, universal nucleic acid testing, school closures, and restrictions on travel and mass gatherings. These measures have curbed the spread of severe acute respiratory syndrome coronavirus 2 (SARS-CoV-2) and led to a decrease in the activity of traditional respiratory infectious diseases such as influenza [1, 2]. Influenza activity was low worldwide between 2020 and 2021. However, a resurgence of influenza activity was identified in late 2021 following the gradual relaxation of COVID-19 restrictions in countries worldwide after the 2020–2021 influenza season [3]. According to data released by the World Health Organization (WHO), the global overall influenza activity in the 2022–2023 season rebounded fiercely, peaking in late 2022, and many indicators of influenza activity were above the levels seen in previous epidemics [4]. In northern China, influenza activity showed distinct seasonal patterns, typically peaking in the winter months before COVID-19 [5]. During the COVID-19 pandemic, China's “zero-COVID” strategy effectively suppressed influenza. Influenza activity during the 2020–2021 and 2021–2022 seasons was either absent or minimal compared to the periods before the COVID-19 pandemic [6, 7]. In the 2022–2023 influenza season, however, an out-of-season rebound in influenza activity was identified in China during the spring months after the relaxation of COVID-19 restriction measures in early January 2023 [8].

Beijing, the capital of China, is located in the temperate zone of northern China, and has the similar characteristics of influenza seasonality pattern of northern China [9]. During the spring months in 2023, a similar pattern of the influenza epidemic trend to that in northern China had been observed in Beijing. In this study, we aimed to quantify the changes in influenza epidemic intensity, epidemic onset and duration, and influenza transmissibility pre- and post-COVID-19 period in Beijing, and to compare the epidemiological characteristics of various influenza virus subtypes in different age groups over the 2 periods. This analysis will hopefully provide evidence to assist the optimization of medical resource allocation, vaccination strategies, and public health policies in the post-COVID-19 period.

## METHODS

### Influenza Surveillance Data Source

In this study, influenza-like illness (ILI) was defined as patients presenting with fever ≥38.0°C and cough or sore throat.

### Influenza-like Illness Surveillance

The numbers of daily and weekly ILI consultations by age group (0–4 years, 5–14 years, 15–24 years, 25–59 years, and ≥60 years) were obtained through the Beijing Monitoring and Early Warning System for Infectious Diseases. This internet-based system is coordinated and managed by the Beijing Center for Disease Control and Prevention (BJCDC). Numbers of attendances and ILIs were entered by outpatients and emergency departments of internal medicine, fever clinics, and pediatrics of 144 secondary and tertiary sentinel hospitals in Beijing [10].

### Influenza Virus Surveillance

Influenza virus surveillance data were collected from the influenza virus surveillance system of Beijing [11], which was expanded from the original 23 sentinel hospitals and 17 collaborating laboratories to 39 sentinel hospitals and 18 collaborating laboratories in February 2023. In this system, each sentinel hospital collected 10 to 20 pharyngeal swab specimens of outpatient ILI cases per week. The collected specimens were transported to collaborating laboratories for real-time reverse-transcription polymerase chain reaction testing of influenza virus subtypes/lineages. Collaborating laboratories provided BJCDC with weekly summaries of test volumes and influenza virus test results. Starting from week 39, 2015, demographic information of the subjects including birth date and sex were recorded while sampling, and was provided to BJCDC by collaborating laboratories. Thus, demographic information used for analysis in this study does not include those before week 39, 2015.

### Data Collection

We defined week 27 of each year to week 26 of the following year as an influenza surveillance year in Beijing. Data of ILI surveillance and influenza virus surveillance in Beijing were collated for the period week 27, 2014 (the week ending 6 July 2014) to week 26, 2020 (the week ending 28 June 2020) and for the period week 27, 2022 (the week ending 10 July 2022) to week 26, 2023 (the week ending 2 July 2023). During an influenza surveillance year, the influenza epidemic period was regarded as an influenza season. In this study, we defined the baseline “pre-COVID-19” period as the 2014–2020 influenza seasons. As the WHO declared the COVID-19 pandemic as a Public Health Emergency of International Concern on 11 March 2020 [12], this period was selected to allow inclusion of 6 full influenza seasons of baseline data. The 2022–2023 influenza season was defined as the “post-COVID-19” period, since this period was after the zero-COVID policy was lifted around 8 January 2023 [13].

### Statistical Analysis

Because influenza-like symptoms can be caused by multiple pathogens other than influenza, we multiplied the daily or weekly ILI number by the weekly influenza-positive rate to obtain the proxy measure of influenza activity (hereafter “daily/weekly ILI^+^”). Weekly ILI^+^ counts by age group were obtained by multiplying the number of weekly ILI in each age group by the weekly positive rate of influenza virus in that age group. The overall incidence rate of influenza per 100 000 population was calculated as the number of weekly ILI^+^ divided by the yearly population of Beijing [14] and multiplied by 100 000. Age-specified incidence was calculated as the number of weekly ILI^+^ by age group divided by the yearly population of different age groups and multiplied by 100 000.

To quantify the onset, duration, and intensity of influenza activity in Beijing, we used the moving epidemic method (MEM) [15, 16] to estimate epidemic threshold and 3 intensity thresholds (low, medium, and high) based on the historical weekly ILI^+^ data from 2014 to 2020. All weekly ILI^+^ data for each influenza surveillance year were included in our analysis. The week in which the weekly ILI^+^ value was higher than the epidemic threshold was considered the first week of the influenza epidemic for that season, and the week before the weekly ILI^+^ value dropped below the epidemic threshold was considered the last week. Each epidemic was classified into 1 of 4 intensity levels (low, medium, high, and very high) according to 3 intensity thresholds—which were defined as the upper limits of the 40%, 90%, and 97.5% 1-sided confidence intervals (CIs) for the geometric means of the 30 highest weekly epidemic rates in 2014–2020.

To assess the difference in influenza transmissibility between the 2 periods, we estimated the daily instantaneous reproduction number (R_t_) of the 2014–2020 and 2022–2023 seasons. To reduce statistical noise from the datasets, 7-day centered moving averages of the daily ILI^+^ values during each influenza epidemic period were used to estimate the daily R_t_ of the corresponding period by using a method adapted from Cori et al [17, 18]. The serial interval was assumed to follow a gamma distribution with a mean of 2.85 days and standard deviation of 0.93 days [19].

Demographic information on ILI cases in which pharyngeal swabs were collected was entered using EpiData Version 3.1. WPS Office was used to create the datasets. Data analysis and plotting were performed using R version 4.0.3 software. Count data were interpreted as the medians, ranges, and interquartile ranges (IQRs). Only the epidemic periods of the influenza surveillance years were included in our comparative analysis. The Wilcoxon rank-sum test was used to compare 2 paired groups, and the Kruskal-Wallis test was used to compare 3 or more groups. Generalized linear model of quasi-Poisson regression was used to measure the changes in incidence rates for the general population and each age group between the 2014 and 2020 and 2022–2023 seasons using different periods (pre- or post-COVID-19) as the variable. Rate ratios (RRs) and 95% CIs were reported as the relative changes in the incidence rates between the pre-COVID-19 period and the 2020–2023 season. MEM was applied using the Moving Epidemic Method Shiny Web Application version 2.16 [20]. The EpiEstim package was used to estimate the R_t_. Statistical significance was set at *P* < .05.

This study was approved by the institutional review board and human research ethics committee of the Beijing Center for Disease Control and Prevention.

## RESULTS

### General Information of Influenza Activity

During the 2014–2020 influenza seasons, a total of 2 352 153 ILI consultations were reported through the Beijing ILI surveillance system, with the annual average ILI number of 392 026. During the 2022–2023 influenza season, a total of 524 574 ILI consultations were reported.

During the 2014–2020 influenza seasons, a total of 45 174 specimens from ILI patients were collected and laboratory tested, and the average positive rate was 37.5% (16 946). Of the positive specimens, 24.3% (4117) were subtyped as influenza A(H1N1), 42.4% (7188) were influenza A(H3N2), and 33.5% (5681) were influenza B. During the 2022–2023 influenza season, a total of 8442 specimens from ILI patients were collected and laboratory tested; the average positive rate was 48.8% (4120). Of these, 47.2% (1944) were subtyped as influenza A(H1N1), 52.7% (2173) were influenza A(H3N2), and 0.1% (3) were influenza B. The characteristics of influenza viruses by subtype and by age group can be seen in Supplementary Figures 1 and 2.

### Epidemic Onset and Duration

The influenza epidemic threshold of weekly ILI^+^ calculated by MEM was 2845.94. According to this standard, the onset weeks of epidemic periods occurred between week 47 to week 1 of the following year for 2014–2020 seasons, and the average epidemic duration of influenza was 18 weeks for the 6 seasons (range, 9–23 weeks). In the 2022–2023 season, the onset week of the epidemic period was week 8, 2023, which was notably later than that of the pre-COVID-19 period. The epidemic duration of influenza was 10 weeks in the 2022–2023 season, shorter than the average epidemic duration of the pre-COVID-19 period (Table 1). During the 2014–2020 seasons, the peak time of influenza epidemic occurred between week 51 and week 6 of the following year, with an average interval of 6 weeks (range, 5–8 weeks) from onset to peak time (Table 1). During 2022–2023, the peak time of influenza epidemic appeared in week 10, 2023, which was only 3 weeks apart from the onset week, shorter than that of the pre-COVID-19 period.

**Table 1.** Time of Onset, Duration, and Peak of Influenza Epidemics, and Maximum Epidemic Intensity Levels for the Influenza Surveillance Years of 2014–2020 and 2022–2023 in Beijing, China

Influenza Surveillance Year	Epidemic Onset (Year-Week)	Epidemic Duration (Weeks)	Maximum Peak^a^ Time (Year-Week)	Duration From Onset to Maximum Peak^a^ Time (Weeks)	Maximum Epidemic Intensity Level
2014–2015	2014-47	21	2014-51	5	Medium
2015–2016	2016-1	17	2016-6	6	Low
2016–2017	2016-46	22	2017-1	8	Low
2017–2018	2017-48	15	2018-1	6	High
2018–2019	2018-50	23	2019-2	5	High
2019–2020	2019-49	9	2020-2	6	Medium
2022–2023	2023-8	10	2023-10	3	Very high

^a^Maximum value of the product of the number of weekly influenza-like illness number and weekly influenza positive rate during an influenza surveillance year.

### Epidemic Intensity

**Figure 1.**
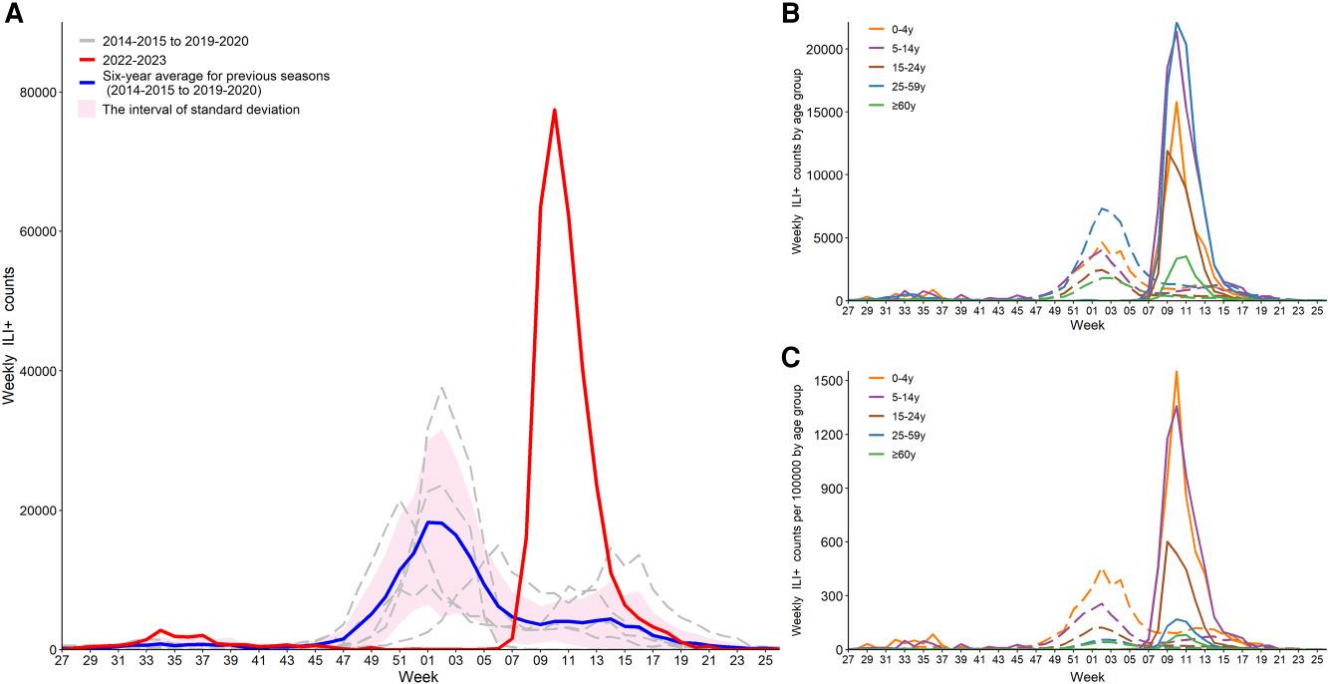
Time series plot of weekly ILI^+^ (proxy value of influenza activity) counts for the influenza surveillance years of 2014–2020 and 2022–2023 in Beijing, China. *A*, Overall weekly ILI^+^ counts of 2 study periods. *B*, Weekly ILI^+^ counts by age group. *C*, Weekly ILI^+^ counts per 100 000 by age group. Dashed lines in (*B*) and (*C*) represent the average ILI^+^ count by age group of the 2014–2020 surveillance years, and solid lines represent the ILI^+^ count by age group of the 2022–2023 surveillance year.

During the 2022–2023 influenza season, the total number of ILI^+^ (308 049) was 1.87 (range, 1.09–3.28) times the annual average ILI^+^ number of 2014–2020 seasons (164 435 [range, 93 951–282 349]). The peak value of weekly ILI^+^ (77 463) was 4.25 (range, 2.06–8.31) times the average peak value of weekly ILI^+^ of the 2014–2020 seasons (18 226 [range, 9322–37 656]) (Figure 1*A*). The overall incidence rate in the 2022–2023 season was 3.35 (95% CI, 2.33–4.83) times that of the 2014–2020 seasons.

**Figure 2.**
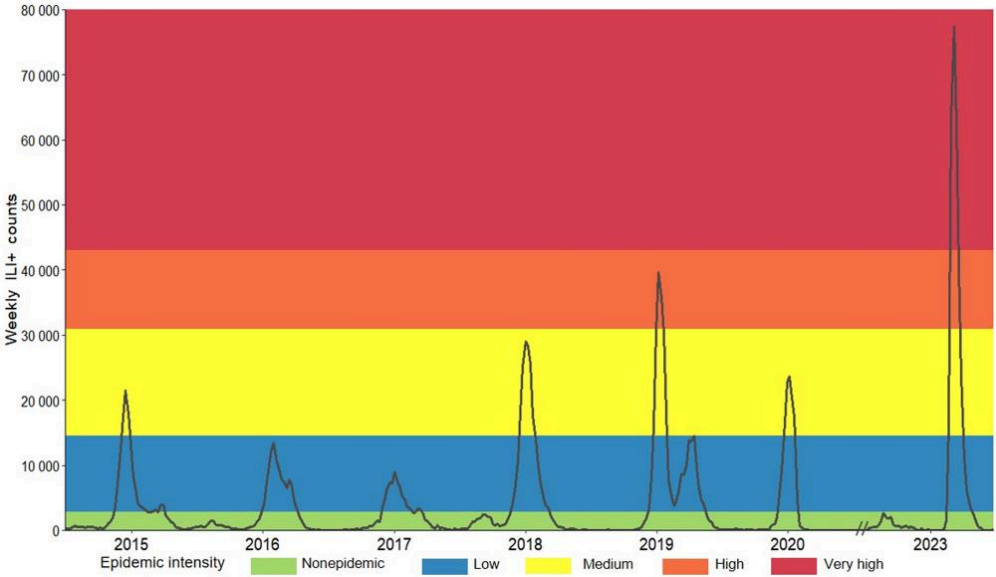
Weekly ILI^+^ (proxy value of influenza activity) counts and influenza epidemic intensity levels for the influenza surveillance years of 2014–2020 and 2022–2023 in Beijing, China.

The intensity thresholds of weekly ILI^+^ for medium, high, and very high epidemic activity by MEM were 14 543.45, 30 868.49, and 43 050.63, respectively (Supplementary Figure 3). According to this standard, the maximum epidemic intensity of the 2022–2023 season reached the very high level, while the highest epidemic intensity in 2014–2020 seasons only reached the high level (Table 1 and Figure 2).

### Influenza Activity by Age Group

**Figure 3.**
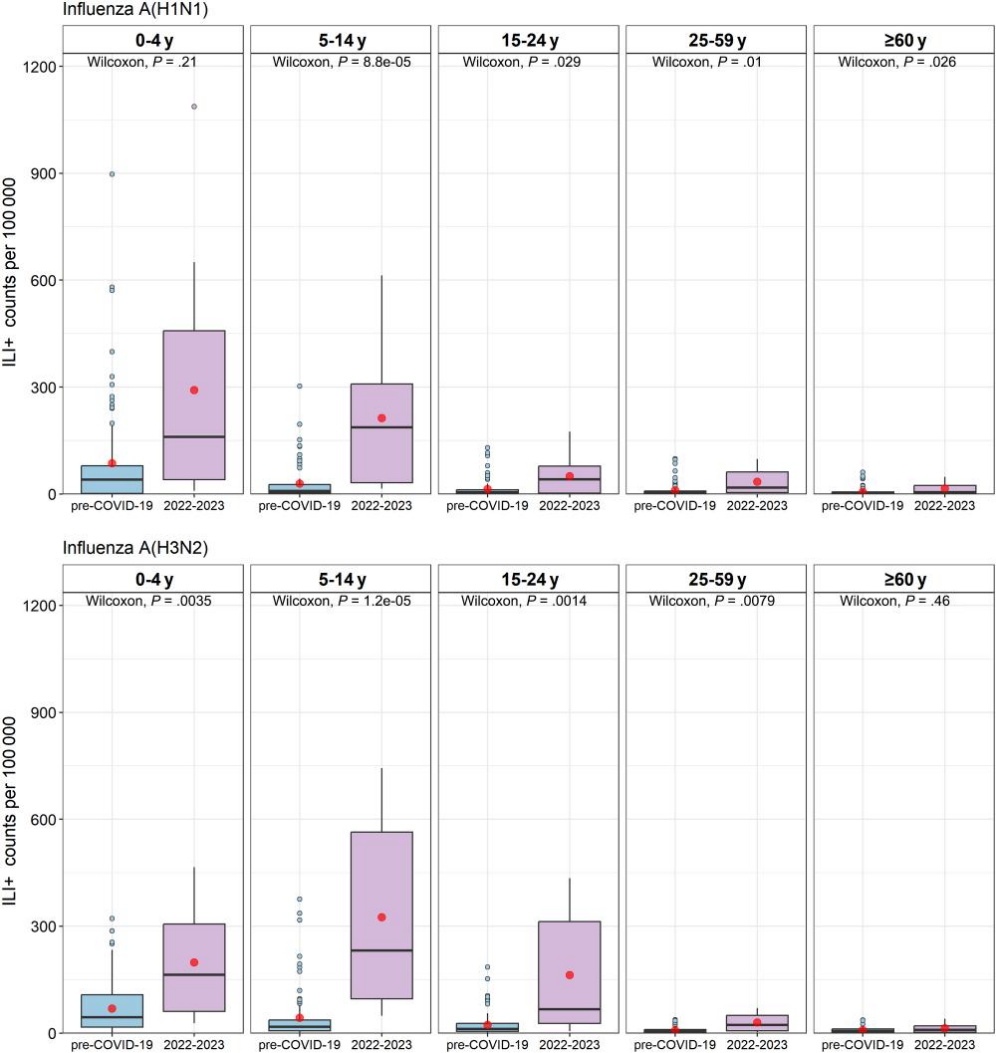
Weekly ILI^+^ (proxy value of influenza activity) counts per 100 000 comparison between pre–coronavirus disease 2019 (COVID-19) and 2022–2023 by age group for influenza A subtypes in Beijing, China. The box plots are based on weeks within epidemic duration. The central bar indicates the median weekly ILI^+^ counts per 100 000, and the mean is shown as red points. The lower and upper bounds of the box indicate the first and third quartiles (interquartile range [IQR]), the lower whisker extends from the first quartile to the lowest value within 1.5 × IQR of the first quartile, and the upper whisker extends from the third quartile to the highest value within 1.5 × IQR of the third quartile. *P* values (Wilcoxon rank-sum test) comparing median of weekly ILI^+^ counts per 100 000 for 2 study periods in each age group are presented at the top of each panel.

As shown in Figure 1*B* and 1*C* and Supplementary Figure 4, the changes of influenza activity before and after the COVID-19 period varied by age group. First, there were differences in the changes in epidemic intensity among age groups of the 2 study periods. As shown in Table 2, the relative increase in incidence rate was highest in the 5- to 14-year-old group (RR, 4.60 [95% CI, 3.19–6.65]), followed by the 15- to 24-year-old group (RR, 4.09 [95% CI, 2.67–6.26]), and the least in the ≥60-year-old group (RR, 1.59 [95% CI, .93–2.74]). For influenza A(H1N1) virus, the median weekly ILI^+^ count per 100 000 of the 2022–2023 season in each age group was significantly higher than that of the pre-COVID-19 period (Figure 3). For influenza A(H3N2), the median weekly ILI^+^ count per 100 000 of the 2022–2023 season in each age group except for the ≥60-year-old group was significantly higher than that of the pre-COVID-19 period (Figure 3). Second, the characteristics of the incidence rate of influenza by age group were different between the 2 study periods. During the pre-COVID-19 period, both the overall average weekly ILI^+^ count per 100 000 and the average weekly ILI^+^ count per 100 000 of influenza A(H1N1) and A(H3N2) were the highest among the 0- to 4-year-old group, and decreased with the increase of age. During the 2022–2023 season, the overall average weekly ILI^+^ count per 100 000 was the highest among the 5- to 14-year-old group, followed by the 0- to 4-year-old group, and lowest in the ≥60-year-old group. This result was similar to the incidence characteristics of influenza A(H3N2) by age group. For influenza A(H1N1), however, the average weekly ILI^+^ count per 100 000 was the highest among the 0- to 4-year-old group, followed by the 5- to 14-year-old group, and lowest in the ≥60-year-old group (Supplementary Figure 5).

**Table 2.** Average Weekly Numbers of ILI^+^ (proxy value of influenza activity) and of ILI^+^ per 100 000 During the 2014–2020 and 2022–2023 Influenza Seasons in Beijing, China

Variable	2014–2020 Influenza Seasons		2022–2023 Influenza Season		RR of No. of ILI^+^ (per 100 000) for 2022–2023 vs 2014–2020 (95% CI)	*P* Value
	Average Weekly No. of ILI^+^ (Min–Max)	Average Weekly No. of ILI^+^ per 100 000^a^ (Min–Max)	Average Weekly No. of ILI^+^ (Min–Max)	Average Weekly No. of ILI^+^ per 100 000^a^ (Min–Max)		
Overall	9220.68 (1091.44–37 655.56)	42.12 (4.99–172.00)	30 804.91 (3256.20–77 463.12)	140.71 (14.87–353.82)	3.35 (2.33–4.83)	<.001
Age group, y^b^						
0–4	2204.07 (269.18–10 578.96)	216.88 (26.49–1040.98)	5241.71 (385.18–15 779.29)	515.79 (37.90–1552.70)	2.38 (1.57–3.64)	<.001
5–14	1906.30 (237.25–7672.81)	121.01 (15.06–487.08)	8743.77 (1026.90–21 370.30)	555.07 (65.19–1356.62)	4.60 (3.19–6.65)	<.001
15–24	1057.92 (14.78–4072.00)	53.32 (0.74–205.24)	4308.05 (165.51–11 911.99)	217.13 (8.34–600.38)	4.09 (2.67–6.26)	<.001
25–59	3273.80 (228.40–15 941.25)	25.15 (1.75–122.45)	8761.71 (505.23–22 123.67)	67.30 (3.88–169.93)	2.69 (1.69–4.27)	<.001
≥60	813.70 (20.48–3490.94)	18.93 (0.48–81.21)	1290.71 (139.99–3528.11)	30.03 (3.26–82.08)	1.59 (0.93–2.74)	.096

Abbreviations: CI, confidence interval; ILI^+^, the product of the number of weekly influenza-like illness number and weekly influenza positive rate; RR, rate ratio.

^a^The total population and population by age group of Beijing used for calculations is from the seventh national population census of China.

^b^Due to the collection of age information on the sampling subjects for pathogen testing starting from week 39, 2015, the ILI^+^ data used for statistical analysis in age group do not include data prior to week 39, 2015.

### Estimated R_t_ of Influenza

**Figure 4.**
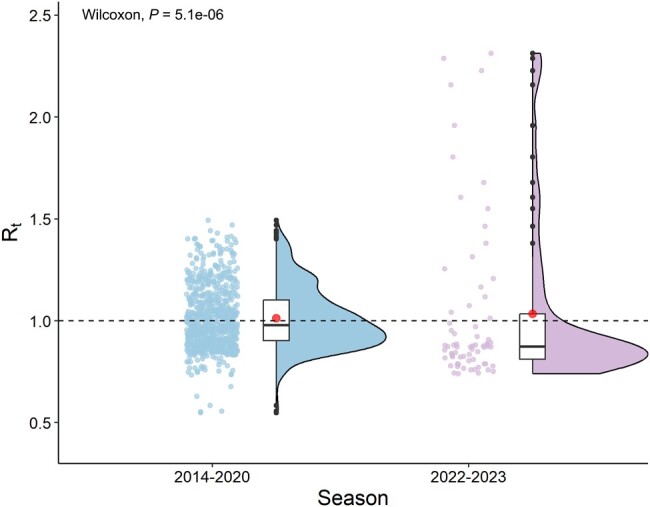
The violin plots of instantaneous reproduction number (R_t_) of the 2014–2020 and 2022–2023 seasons in Beijing, China. The central bar, box, and whiskers of the box plots within the violin plots indicate the median R_t_ value, the interquartile range, and the minimum and maximum, respectively. The mean is shown as red points. Jitter plots indicate the distribution of R_t_ value. *P* values (Wilcoxon rank-sum test) comparing median R_t_ value for 2 study periods are presented at the top of each panel.

As shown in Figure 4, both the value and the distribution of R_t_ of influenza appeared to be different between the 2 study periods. The maximum R_t_ value (2.31) of the 2022–2023 season was much higher than the average maximum R_t_ value (1.35 [range, 1.24–1.49]) of the pre-COVID-19 period, while the median R_t_ value (0.87 [IQR, 0.82–1.30]) of the 2022–2023 season was significant lower than the average median R_t_ value (0.98 [IQR, 0.92–1.10]) of the pre-COVID-19 period (*P <* .001). Time and numerical distribution of R_t_ value of influenza seasons can be seen in Supplementary Figures 6 and 7.

## DISCUSSION

This study analyzed the changes of influenza activity in terms of the timing and intensity before and after the COVID-19 period in Beijing. The results showed that Beijing experienced an intense out-of-season epidemic in the 2022–2023 influenza season with the dominant influenza virus subtypes of A(H1N1) and A(H3N2). Age group–stratified analysis showed that the highest incidence rate was among school-aged children aged 5–14 years in the 2022–2023 season, while the highest incidence rate was among preschool children aged 0–4 years in the pre-COVID-19 period.

The out-of-season epidemic pattern of influenza during the 2022–2023 season in Beijing was typical for northern China [21]. However, this did not seem to be a global trend in terms of influenza epidemics, as the epidemic patterns varied significantly by country. Compared to other temperate countries in the Northern Hemisphere, the 2022–2023 influenza season in Beijing began much later and was more active. Surveillance reports from North American and European countries showed that the influenza activity during the 2022–2023 season peaked in late 2022, relatively earlier than it did during the pre-COVID-19 influenza seasons [22–25]. The activity levels in these countries were slightly higher than the average level before the pandemic but did not exceed the peak of the high-severity seasons. Japan and Korea, in contrast, experienced a prolonged spread of infections in the 2022–2023 season, with a record-high influenza activity in the late spring and early summer seasons [26, 27].

The variations in influenza epidemic patterns in different countries may be related to the different durations and levels of stringency of COVID-19 restrictions measures in each one. It has been shown that stricter adherence to nonpharmaceutical interventions (NPIs) or restrictions correlates with a more thorough easing of NPIs or restrictions, ultimately leading to more severe influenza rebounds [28, 29]. Beijing, following the national COVID-19 prevention and control strategy, implemented China's zero-COVID policy for about 3 years, which was longer and stricter compared to the policies of many other countries. While NPI or restriction measures suppressed COVID-19 effectively and consistently, they led to a longer interruption of the circulation of other respiratory viruses—such as the influenza virus. Low levels of long-term exposure to the influenza virus may have given rise to more susceptible individuals and caused a stronger rebound in influenza activity during the periods following the easing of COVID-19 restrictions [1, 30].

Our age-specific analysis showed a significantly higher incidence of influenza among children under the age of 14 in the 2022–2023 season with the highest incidence observed in school-aged children (5–14 years), suggesting a greater impact of the interruption of influenza circulation during the COVID-19 pandemic on children. This rebound in influenza activity among children may have been caused by a combination of multiple factors. On the one hand, the prolonged absence of influenza virus circulation can reduce a population's immunity to influenza viruses, especially among young children with few pre-COVID-19 exposures. On the other hand, school closures during the COVID-19 pandemic and the overlapping vaccination times of the COVID-19 and influenza vaccines may have led to a decline in the influenza vaccination rate among school-aged children compared to previous years. In addition, the reopening of schools and kindergartens after the relaxation of COVID-19 restrictions promoted the spread of influenza viruses among children. Furthermore, since the second semester of the 2022–2023 school year started on 13 February 2023 (the starting date of week 7) [31], coinciding with the rapidly increasing period of influenza activity, it is reasonable to presume that children may have played a dominant role in boosting the community spread of the influenza virus during its 2022–2023 epidemic wave. Studies have reported that healthy adults who work with children, or those with young children at home, are >1.5 times more likely to be exposed to influenza viruses [32–34] and that children in preschool and primary school may play important roles as driving forces of influenza epidemics in the community [35]. Müller and colleagues suggested that school-aged children might play a more critical role in the transmission network of the influenza virus than preschool-aged children [36].

By further analyzing the incidence of influenza across subtypes, we observed that the incidence of influenza A(H1N1) among children aged 0–4 years was higher than that in other age groups, both before and after the COVID-19 period. We observed that the incidence of influenza A(H3N2) was highest in children aged 5–14 years during the 2022–2023 season, rather than in children aged 0–4 years (which was the case in the pre-COVID-19 period). This may be partially explained by the findings of Ranjeva et al, which showed that protective immunity against circulating strains of influenza A viruses waned to half of its peak level 3.5–7 years following infection, and waned faster against influenza A(H3N2) than A(H1N1) [37]. In addition, the effectiveness of vaccination against influenza A(H1N1) is generally higher than that against influenza A(H3N2) [38, 39]. Therefore, the level of preexisting immunity against influenza A(H3N2) acquired through infection or vaccination may be lower than that against influenza A(H1N1) among children aged 5–14 years after the COVID-19 pandemic, resulting in a higher incidence of influenza A(H3N2) in the 5- to 14-year-old group. However, results regarding the transmissibility of influenza A(H1N1) and A(H3N2) have been inconsistent across different studies [40, 41], and the complex immune responses necessary for protection against influenza viruses are still not fully understood. Thus, further long-term surveillance and research are warranted in the future.

We estimated the R_t_ values for the 2014–2020 and 2022–2023 seasons, to quantitatively compare the maximum real-time transmissibility at peak times and the average transmissibility of influenza pre- and post-COVID-19 period. We observed that the estimated maximum value of R_t_ for the 2022–2023 season was higher than that seen in previous seasons in Beijing, whereas the median R_t_ value was significantly lower. Our findings quantitatively demonstrate that Beijing experienced a shorter and more intense wave of influenza epidemics during the 2022–2023 season, indicating a higher transmission potential of influenza, which suggests the need to strengthen surge capacity in the public health system in the future.

The significance of our study is that we assessed the epidemic characteristics of influenza before and after the COVID-19 period both qualitatively and quantitatively, providing comprehensive insights into the changes in influenza activity in Beijing. Our findings highlight the necessity for continuous influenza surveillance and timely assessments of influenza activity and the implementation of targeted control measures—such as improving protective measures for susceptible populations and optimizing vaccination strategies.

This study was subject to several key limitations worth noting. First, it analyzed only 1 post-COVID-19 influenza season. It is unclear whether this high-intensity spring epidemic in Beijing has sufficiently reduced the susceptible population to pre-COVID-19 levels, and how long it will take for normal winter influenza seasonality to resume. A recent modeling study predicted an increasing ILI burden over the next 3 years in both northern and southern China [42]. These findings highlight the need to prepare for potentially large influenza outbreaks in the future. Second, the study period preceding COVID-19 included the data from the 2019–2020 influenza surveillance years. To contain the COVID-19 outbreak, Beijing launched a first-level public health emergency response at the end of January 2020, which may have shortened the influenza epidemic duration and lowered the average epidemic duration in that year compared to previous ones. In addition, owing to the lack of demographic information on ILI sampled before week 39 of 2015, the results of our age-specific analysis may have been biased compared to our results for the overall population. However, the epidemiological characteristics of influenza by age group were relatively similar in each season of the pre-COVID-19 period, and our results reflect the age-specific characteristics of influenza activity in the pre-COVID-19 period. Third, our study did not analyze the epidemic intensity by different subtypes of influenza. Last, the study did not analyze the possible differences in spatial trends in influenza activity pre- and post-COVID-19 in Beijing; therefore, further exploration is warranted on this topic.

## CONCLUSIONS

In conclusion, our study reported a strong rebound of influenza activity in the 2022–2023 season after the relaxation of COVID-19 restrictions in Beijing, China, characterized by an unusually late onset, much higher intensity, and shorter epidemic duration than the pre-COVID-19 period. This out-of-season epidemic pattern was typical in northern China during this season, but inconsistent with the epidemic trend internationally. Our findings suggested a greater impact of the interruption of influenza circulation during the COVID-19 pandemic on children under the age of 14, who may play a central role in boosting the spreading of influenza virus in the community in the 2022–2023 epidemic wave. Due to the uncertainty of influenza activity patterns in the post-COVID-19 era, maintaining continuous surveillance of influenza activity is very important for examining changes in periodicity and age distribution, in order to develop more targeted public health strategies such as age- and subtype-specific prevention and control measures and vaccination campaigns in the future.

## Supplementary Material

ofae163_Supplementary_Data

## Supplementary Data

Supplementary materials are available at *Open Forum Infectious Diseases* online. Consisting of data provided by the authors to benefit the reader, the posted materials are not copyedited and are the sole responsibility of the authors, so questions or comments should be addressed to the corresponding author.

## Note

***Potential conflicts of interest*.** All authors: No reported conflicts.
