# Supplementary material for: An Intense Out-of-Season Rebound of Influenza Activity After the Relaxation of Coronavirus Disease 2019 Restrictions in Beijing, China
Source: Open Forum Infect Dis. 2024 Mar 20;11(4):ofae163. doi: 10.1093/ofid/ofae163 (PMC10995958; doi:10.1093/ofid/ofae163)
Supplement: ofae163_Supplementary_Data [file ofae163_supplementary_data.docx]

# **An intense out-of-season rebound of the influenza activity after the relaxation of COVID-19 restrictions in Beijing, China**

**Supplementary Figures**


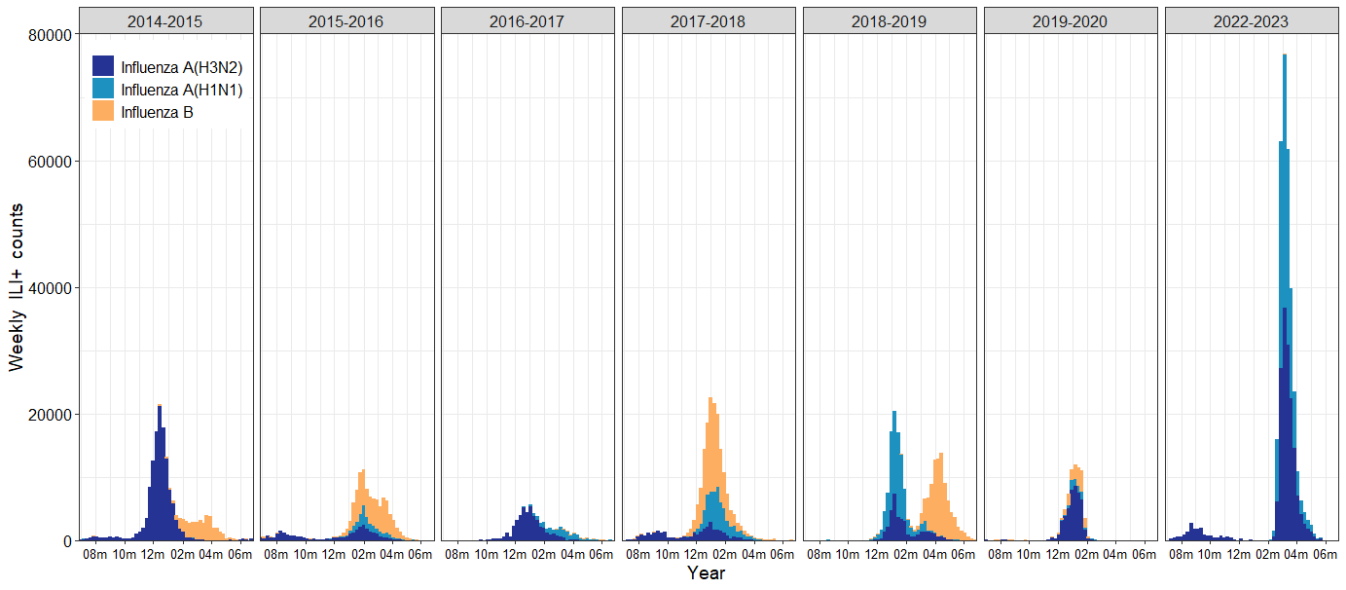


**Figure S1.** Weekly distribution of ILI+ counts by influenza virus subtypes for the influenza surveillance years of 2014-2020, and 2022-2023 in Beijing, China.


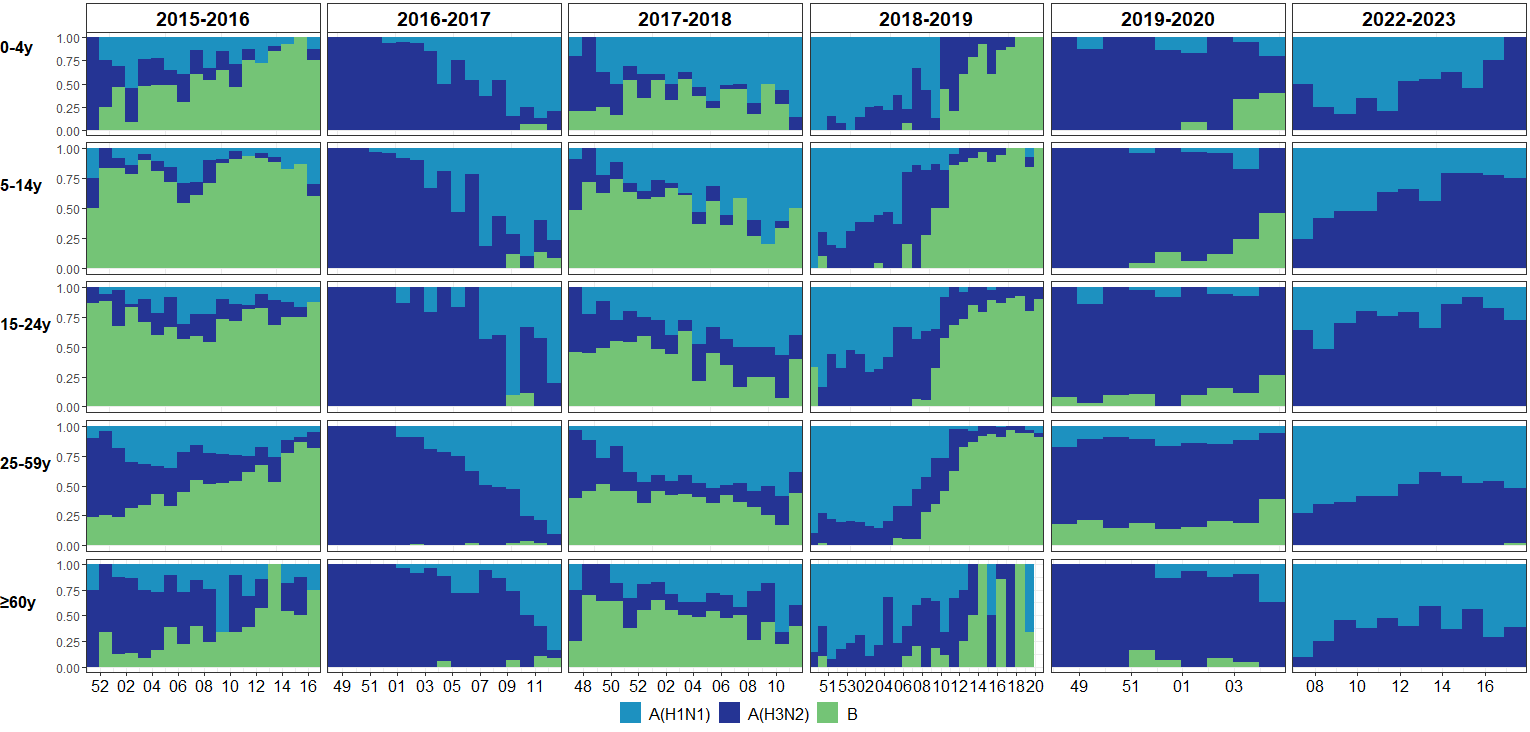


**Figure S2.** Proportion of different influenza virus subtypes by age group in different influenza seasons of 2015-2020, and 2022-2023 in Beijing, China.


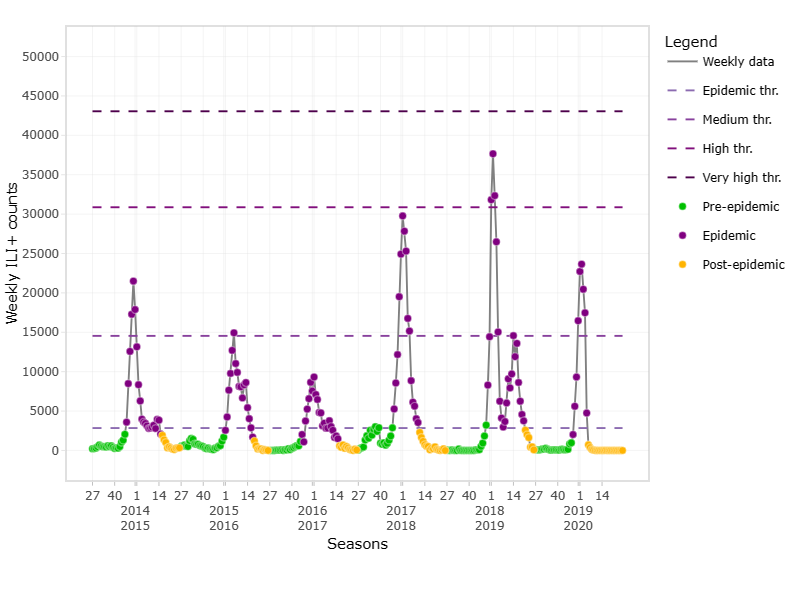


**Figure S3.** Time series of weekly ILI+ counts, the epidemic periods modelled by MEM, and the epidemic threshold, levels of intensity during 2014-2020 in Beijing, China.


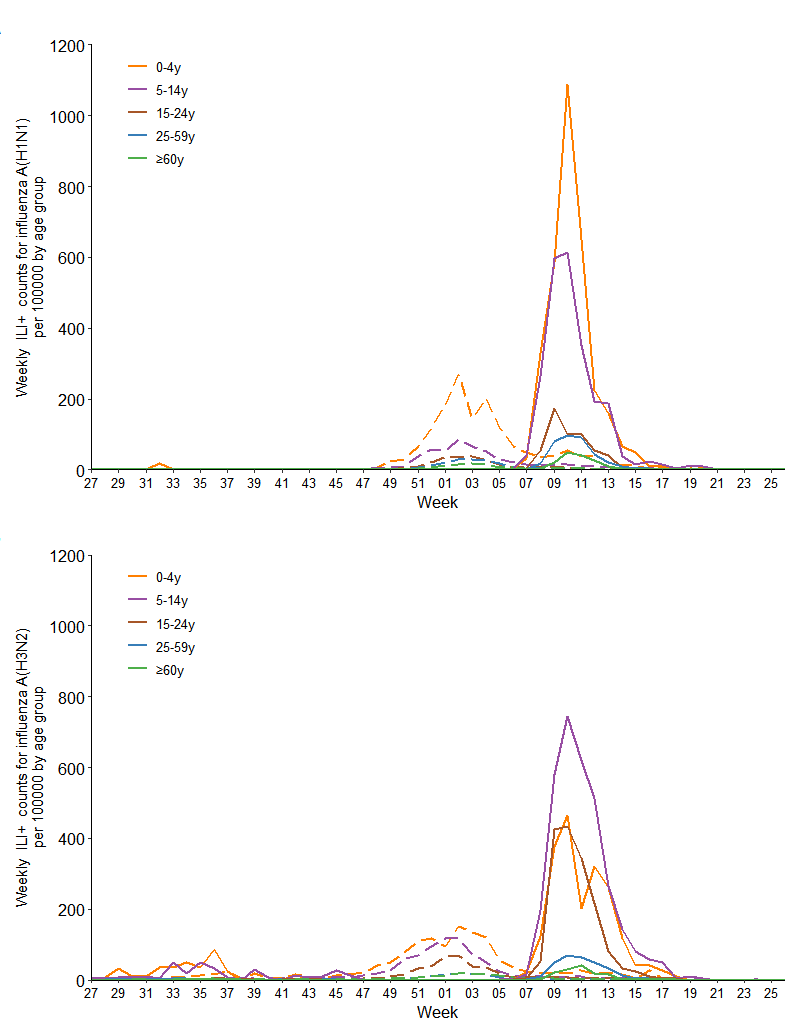


**Figure S4.** Time series plot of weekly ILI+ counts of different influenza A subtypes by age group for the influenza surveillance years of 2014-2020, and 2022-2023 in Beijing, China. ILI+: proxy value of influenza activity. Dashed lines represent the average ILI+ counts of the 2014-2020 surveillance years, and solid lines represent the ILI+ counts of the 2022-2023 surveillance year.


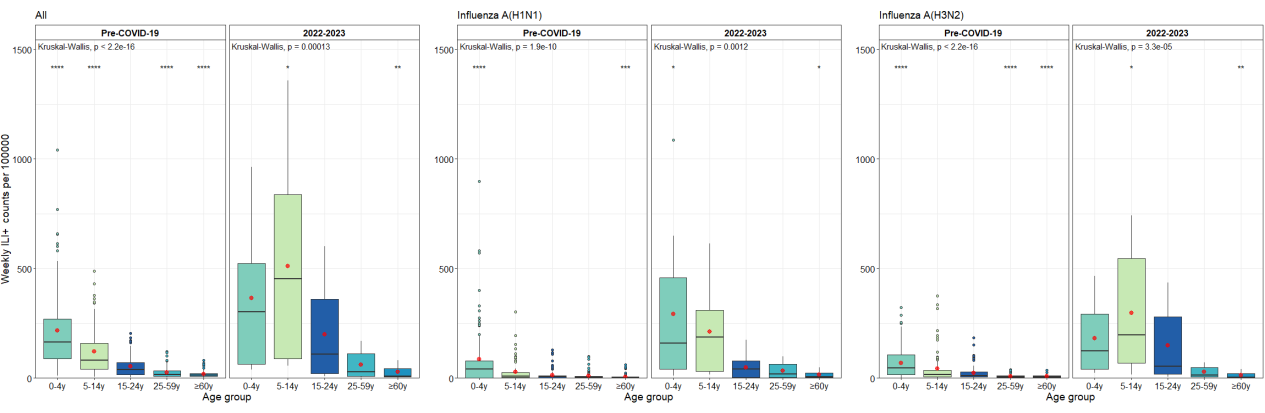


**Figure S5.** Weekly ILI+ counts per 100,000 comparison between age groups and time periods for all influenza virus and for influenza A subtypes during 2014-2020 influenza seasons and 2022-2023 influenza season in Beijing, China. The box plots are based on weeks within epidemic duration. The central bar indicates the median weekly ILI+ counts per 100,000, and the mean is shown as red point. The lower and upper bounds of the box indicate the first and third quartiles (IQR), the lower whisker extends from the first quartile to the lowest value within 1.5*IQR of the first quartile, the upper whisker extends from the third quartile to the highest value within 1.5*IQR of the third quartile. P values (Kruskal-Wallis test) comparing median of weekly ILI+ counts per 100,000 for each age group with all age groups are presented at the top of each panels. * above the bars indicates statistical significance of the analysis results.*0.01 < P ≤ 0.05; **0.001 < P ≤ 0.01; ***P ≤ 0.001.


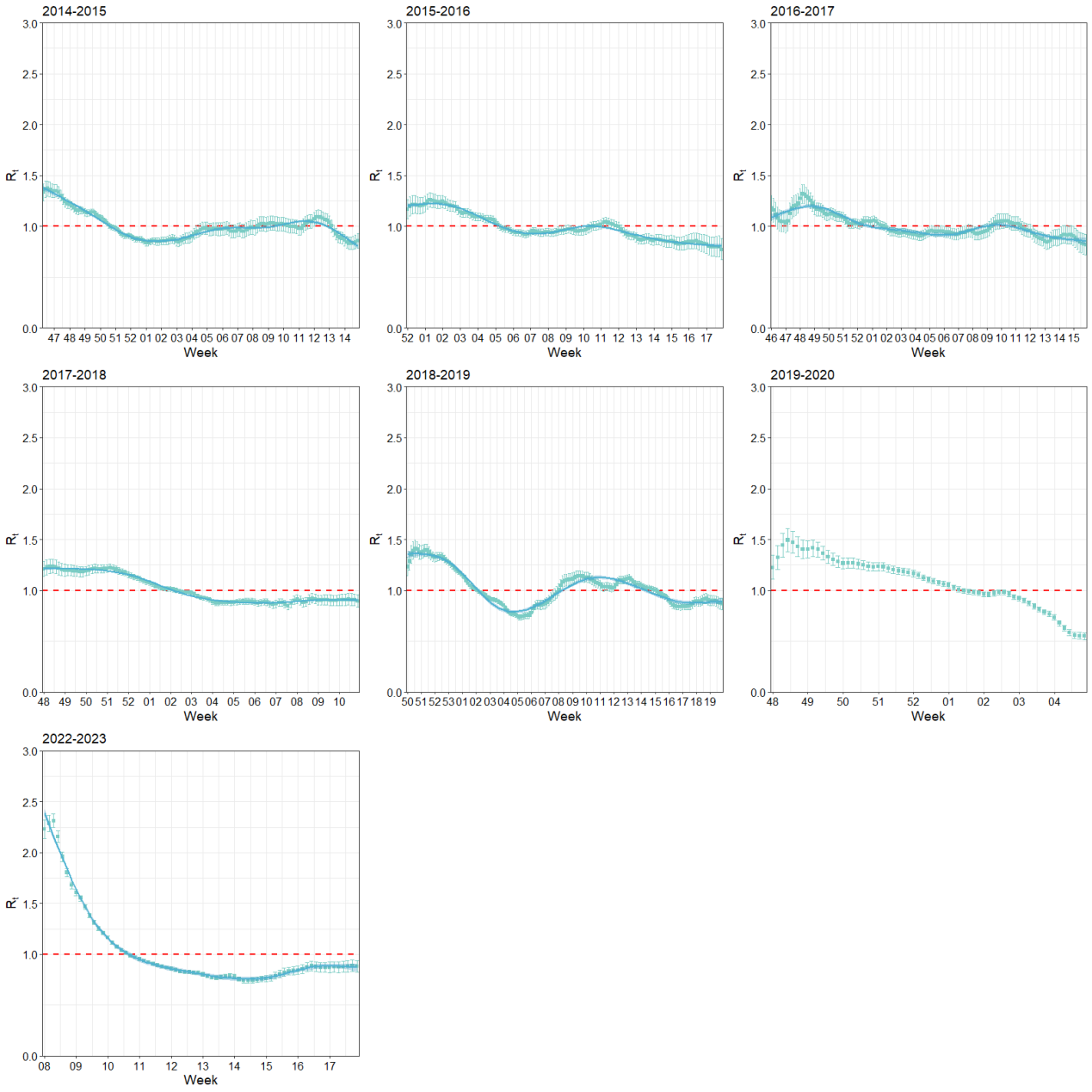


**Figure S6.** Instantaneous reproductive number (R_t_) of influenza during epidemic periods of 2014–2020 and 2022-2023 in Beijing, China. Green dots and vertical solid lines: estimated daily R_t_ and its 95% confidence interval (CI); Blue lines and the blue shade: smoothed daily R_t_ and its 95% CI.


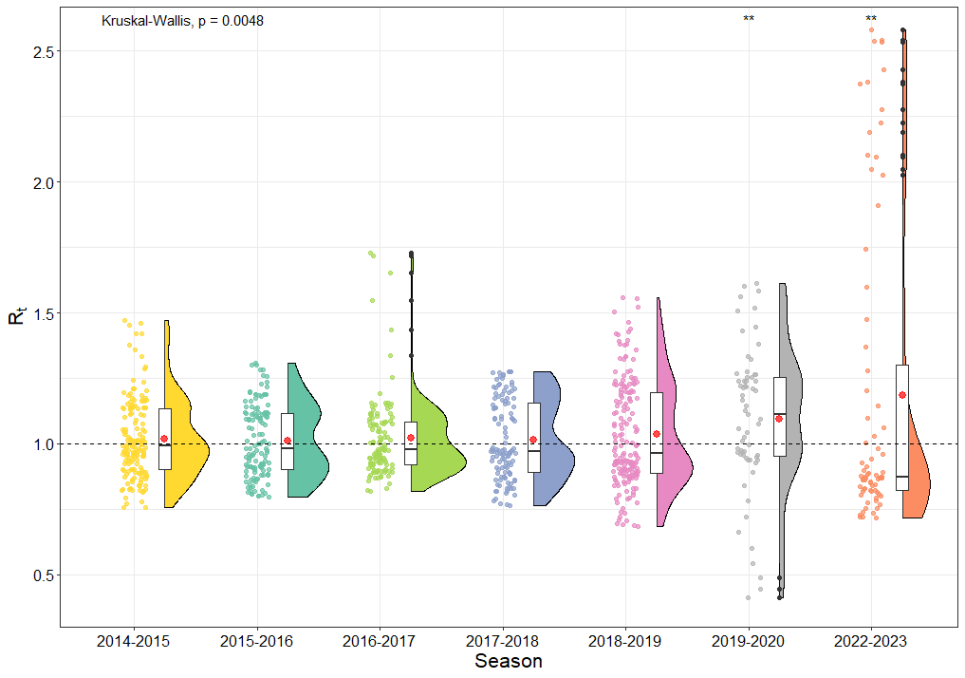


**Figure S7.** The violin plots of instantaneous reproduction number (R_t_) of 2014-2020 seasons and 2022-2023 season in Beijing, China. The central bar , box and whiskers of the box plots within the violin plots indicate the median R_t_ value, the interquartile range and the minimum and maximum, respectively. The mean is shown as red point. Jitter plots indicate the distribution of R_t_ value. P values (Wilcoxon test) comparing median R_t_ value for two study periods are presented at the top of each panels.
